# Integrated analysis of miRNA and mRNA expression profiles in testes of Landrace and Hezuo boars

**DOI:** 10.3389/fvets.2022.942669

**Published:** 2022-10-18

**Authors:** Bo Zhang, Zunqiang Yan, Yi Gao, Jiyou Li, Zike Wang, Pengfei Wang, Qiaoli Yang, Xiaoyu Huang, Shuangbao Gun

**Affiliations:** ^1^College of Animal Science and Technology, Gansu Agricultural University, Lanzhou, China; ^2^Jilin Rongtai Agricultural Development Co., Ltd., Changchun, China; ^3^Gansu General Station of Animal Husbandry Technology Extension, Lanzhou, China; ^4^Gansu Research Center for Swine Production Engineering and Technology, Lanzhou, China

**Keywords:** precocious puberty, small RNA-Seq, microRNA, Hezuo boar, testis

## Abstract

Precocious puberty is closely related to testicular development and spermatogenesis, and there is increasing evidence that miRNAs are involved in regulation of testicular development and spermatogenesis. However, little is known about the regulation of microRNAs (miRNAs) during precocious maturation in Hezuo (HZ) boars. In this study, serum Testosterone (T), Estradiol (E_2_), Follicle-stimulating hormone (FSH) and Luteinizing hormone (LH) levels were detected in HZ and Landrace (LC) boars in the postnatal period at 30, 90, 120, 180, and 240 days, and the testes of HZ and LC boars at 30 and 120 days were used for histological observation. In addition, we performed small RNA-Seq to identify miRNA at sexual immaturity (30-days-old) and maturity (120-days-old) of HZ boar testis (using LC boar as control) to reveal the key miRNA in regulation of precocious puberty. Hormone assay results showed that high levels of T, E_2_, FSH, and LH may be related to precocious sexual maturity of HZ boars, and that FSH may play an important function before sexual maturity. Histological observation showed that HZ boars developed earlier than LC boars and had reached sexual maturity at 120 days. Small RNA-Seq yielded a total of 359 exist miRNAs, 767 known miRNAs and 322 novel miRNAs in 12 samples; 549, 468, 133, and 247 differentially expressed (DE) miRNAs were identified between Ha vs. Hb, La vs. Lb, Ha vs. La, and Hb vs. Lb (log_2_ fold change >1 and *p* < 0.05). Enrichment analysis showed that target genes of these DE miRNAs were enriched in many gene ontology (GO) terms and Kyoto Encyclopedia of Genes and Genomes (KEGG) signaling pathways (such as PI3K-Akt, Hippo and Rap1 signaling pathways) were related to testicular development and spermatogenesis. Further screening, some miRNAs (such as ssc-miR-29b, ssc-miR-199b, ssc-miR-383, ssc-miR-149, ssc-miR-615, and ssc-miR-370) were possibly associated with precocious puberty. These results provide new light on miRNA regulatory mechanisms involved in precocious puberty.

## Introduction

Sexual maturation time is an important indicator of pig reproductive performance, and different breeds reach sexual maturity at different ages. Compared to foreign introduced breeds, some native pig breeds have excellent traits of early maturity that could be inherited stably during the evolutionary process. For example, Ding et al. (1) found that Chinese Meishan boars had appeared all levels of spermatogenic cells in the testicular spermatogenic tubules at 75 days of age, while Duroc boar did not appear. Paixao et al. studied the testicular development of the Bísaro pig, a local Portuguese pig breed, and found that mature complete spermatogenesis appeared at 90 days of age and had sexual capacity between 3.5 and 4.4 months of age, indicating that this breed is characterized by sexual precocity (2). In short, sexual precocity can improve the pig utilization rate, but also shorten the generation interval of excellent pig breed and accelerate the genetic breeding process. Therefore, shortening the time to sexual maturity is important to improving the reproductive efficiency of pigs.

Testosterone (T), produced primarily by the Leydig cells of the testes, has long been known to be the dominant sexual steroid hormone in males and is essential for Sertoli cells prior to testicular maturation (3, 4). However, Leydig, Sertoli, and Germ cells also produce estrogen at various stages of testes development (5). Estrogen contributes to early testicular development and is mainly produced by Sertoli cells in the immature testis (6). Prepubertal goats have considerable amounts of Estradiol (E_2_) in their serum, for example, their testes synthesize estrogen and regulate spermatogenesis during this period (7). Furthermore, testicular development also requires gonadotrophin support, such as follicle-stimulating hormone (FSH) and luteinizing hormone (LH). FSH is a critical regulator of reproductive physiology which promotes testicular Sertoli cell synthesis and stimulates spermatogenesis. In many species [such as rats (8), mice (3), sheep (9), and bulls (10)], FSH secretion occurs in a wave-like pattern and leads to a similar pattern of Sertoli cell development. These findings suggest that high levels of FSH play an important role in early Sertoli cell establishment, which may affect the timing of sexual maturation in animals. LH promotes the growth of testicular Leydig cells and synthesizes testosterone, which also promotes spermatogenesis (11). Therefore, the study of sex hormone secretion level and its change pattern are important to understand development of male mammalian sexual maturity.

microRNAs (miRNAs) are types of small, endogenous, non-coding RNA with ~22 nucleotides in length. It is well–known that miRNA can be bound to the 3'-untranslated region (3'-UTR) by perfect or imperfect complementary of target genes in mammals to inhibit or degrade the translation of mRNA (12). Testis is an important reproductive endocrine organs in male mammals and is the site of testosterone production and spermatogenesis (13). Spermatogenesis is a major biological process in development and differentiation of germ cells in the spermatogenic tubules of the mammalian testis, which is tightly regulated at both the transcriptional and post-transcriptional levels by testicular stage-specific gene expression (14). Thus, identifying key regulators in testis development and spermatogenesis will provide a useful reference for studying the mechanism of precocious puberty. Previous studies have revealed that miRNA is an important regulator for testis development or spermatogenesis by targeting mRNA with important functions (15). For instance, Huang et al. found that miR-375 and miR-499 were differentially expressed in testicular tissues of Junmu No.1 pigs at 1 and 6 month ages (16), Ding et al. identified miR-423 from the testes of Meishan boars at 20, 75, and 270 days ages (17). These miRNAs may play important roles in testicular development or spermatogenesis by regulating their target genes, respectively. In addition, miRNA have important regulatory roles in the synthesis and secretion of reproductive hormones. miR-1197-3p can regulate the synthesis and secretion of *T* in goat testis Leydig cells by targeting *PPARGC1A* (18). Yao et al. found that miR-224 can regulate the TGF-β signaling pathway by regulating *SMAD4* and promote *CYP19A1* gene expression to affect the secretion of E_2_ in mouse (19). However, miRNA in the testis of Hezuo pig has not been investigated.

Hezuo (HZ) pig is a very valuable genetic species resource, which is the unique native pig breed in Qinghai-Tibet Plateau of China, mainly distributed in Xiahe, Zhuoni, and Diebu of Gannan Tibetan Autonomous Prefecture (20). Compared to western breed (such as Landrace and Yorkshire), HZ pig has small size, strong disease resistance and early sexual maturation. HZ boar has sexual desire at 45 days and reach sexual maturity at around 4 months (21). However, there are few reports on precocious puberty in HZ pig. In the study, serum hormones (T, E_2_, FSH, and LH) were assayed in HZ and Landrace (LC) boars at 30, 90, 120, 180, and 240 days. And 12 small RNA-Seq libraries were constructed and sequenced using Illumina-HiSeq™ 2,500 to identify miRNA from Tests of 30-days-old and 120-days-old HZ and LC boars, so as to explore key miRNAs that regulated early maturation process in HZ boar.

## Materials and methods

### Ethics statement

This study followed strictly the animal ethical standards and regulations of the College of Animal Science and Technology, Gansu Agricultural University (approval number 2006–398). All works were made to minimize pain experienced by the experimental boars.

### Animals and determination of sex hormone concentration

In this study, 15 Hezuo and 15 Landrace boars (All boars from the Gansu Sunxiang breeding Co., Ltd. of Zhuoni in China) were used, divided in 5 periods (30, 90, 120, 180, and 240d) of 3 boars each. Blood samples of all 30 boars were drawn from the anterior vena cava. These samples were centrifuged (3,000 rpm for 10 mins) within 1 h of collection, stored at−20°C until assay for T, E_2_, FSH, and LH concentrations. The serum concentrations of T, E_2_, FSH, and LH in each boar was determined in triplicate by a commercial enzyme-linked immunosorbent assay (ELISA) kit (MEIMIAN, Jiangsu, China). The serum levels of T, E_2_, FSH, and LH were measured according to the vendor's instructions. The data were analyzed by one-way Analysis of Variance (ANOVA) using SPSS software.

### Paraffin embedding and HE staining

Testicular samples were fixed in Bouin' solution for 72 h and then were processed into paraffin sections of 4–6 μm thickness, and subjected to hematoxylin and eosin (HE) and examined with a light microscope. Diameter of spermatogenic tubule was measured and the number of spermatogenic cells and Sertoli cells was counted. One-way ANOVA was performed using SPSS software.

### Testis sample collection and RNA extraction

All 30-days-old (sexually immature) and 120-days-old (sexually mature) HZ boars, and LC boars at the same age were castrated to obtain the testes samples. The testes on the same side were immediately frozen in liquid nitrogen and then stored at−80°C until further use. Total RNA was extracted from pooled samples by Trizol reagent kit (Invitrogen, Carlsbad, CA, United States) according to the manufacturer's instructions. Quantity and integrality of total RNA was detected by Agilent 2,100 (Agilent, United States) and NanoDrop 2,000 (Thermo Scientific, United States) instruments, and small RNA obtained from total RNA was used to construct small RNA libraries were then tested for quality and yield using Agilent 2,100 and ABI StepOnePlus Real-Time PCR System (Life Technologies). RNA with a RIN ≥7 was used to construct small RNA libraries and subsequent sequencing. The average RIN value of the RNA samples extracted from all testis tissues was 8.4 ± 0.70.

### Testis sample collection and RNA extraction

12 small RNA libraries were constructed using the NEBNext^®^ multiplex small RNA library prep kit for illumina (#E7, 300). After total RNA was extracted, the RNA molecules in a size range of 18–30 nt were enriched by Polyacrylamide gel Electrophoresis (PAGE). Then the 3' Adapters and 5' adapters were ligated to the RNAs. The ligation products were reverse transcribed by PCR amplification and the 140–160 bp size PCR products were enriched to generate a small RNA library and sequenced using illumina-HiSeq™ 2,500 by gene Denovo Biotechnology Co. (Guangzhou, China). The raw datas generated were deposited in NCBI GEO (GSE192462).

### Sequencing data analysis

In this study, a total 12 small RNA libraries (Ha1, Ha2, Ha3, Hb1, Hb2, Hb3, La1, La2, La3, Lb1, Lb2, and Lb3) for sequencing. Ha, Hb, La, and Lb represent 30d Hezuo boar, 120d Hezuo boar, 30d Landrace boar and 120d Landrace boar, respectively. The sequencing raw reads were filtered by fastp (V0.18.0) (22) to remove low quality reads, adaptor sequences and reads under 18 nt in length. To identify the miRNAs, all of the clean tags were aligned with small RNAs in GenBank database (http://blast.ncbi.nlm.nih.gov) and Rfam database (http://sanger.ac.uk/software/Rfam) to identify and remove non-coding RNAs (ncRNAs), including ribosomal RNA (rRNA), small cytoplasmic (scRNA), small nucleolar RNA (snoRNA), small nuclear RNA (snRNA), and transfer RNA (tRNA). Meanwhile all of the clean tags were also aligned with the reference genome (*Sus scrofa* 11.1).Those mapped to exons or introns might be fragments from mRNA degradation, so these tags were removed. The tags mapped to repeat sequences were also removed. The remaining clean tags were further compared with the mature miRNAs in miRBase 22.1 (http://www.mirbase.org/) using bowtie (23) software. All tags that were not annotated to miRBase 22.1, which matched the pig genome assembly were used to predict novel miRNAs according to their genome posi-tions and hairpin structures predicted by software miRDeep2 (24), and miRDeep2 was used to quantify the reads that aligned to known mature miRNAs. The following priority rules were used to ensure that each small RNA has a unique and accurate anno-tation results: exist miRNA > known miRNA > rRNA > tRNA > snRNA > snoRNA > scRNA > novel miRNA.

### Differentially expressed (DE) miRNA analysis and RT-qPCR validation

Total miRNA consists of exist miRNA, known miRNA and novel miRNA. Expression levels of the miRNA were first normalized to obtain the expression levels of transcripts per million (TPM) as the following formula: TPM = Actual miRNA counts/Total counts of clean tags × 10^6^ (25). In addition, we filter out the miRNA whose TPM is <1 for PCA analysis. The heatmap of total miRNA was drawn to display the expression levels of miRNA in different groups and to cluster miRNA with similar expression pattern. miRNA differential expression analysis was performed by edgeR (26) software between four different groups. We identified miRNA with a log_2_ fold change > 1 and *p* < 0.05 in a comparison as significant DE miRNA.

To validate datas accuracy, 10 differentially expressed miRNAs were randomly selected for real-time quantitative PCR (RT-qPCR) analysis. These miRNAs included ssc-miR-149, ssc-miR-217, ssc-miR-31, miR-383-x, miR-29-y, miR-93-y, miR-409-y, novel-m0010-5p, novel-m0003-5p, and novel-m0042-3p. The primer sequences of the selected miRNAs were designed and are listed in Supplementary Table 1, and house-keeping gene U6 was used as an endogenous control. Total RNA was reverse-transcribed to cDNA using the miRNA 1st strand cDNA synthesis kit (Accurate Biotechnology, Hunan, China). Synthesized cDNA was used to perform RT-qPCR as template using the 2 × SYBR^®^ Green Pro Taq HS Premix II (Accurate Biotechnology, Hunan, China). RT-qPCR was performed in a LightCycler 480 II instrument (Roche, Basel, Switzerland) and the thermal cycler program included an initial denaturation at 95°C for 3 mins, followed by 40 cycles at 95°C for 15 s; 57°C for 15 s; and 72°C for 20 s. miRNA expression was quantified relative to U6 expression using the 2^−ΔΔCt^ method (27).

### Target gene prediction and enrichment analysis of the differentially expressed miRNAs

The functions of miRNAs on different physiological activities can be achieved through their binding to corresponding target mRNAs. The prediction and functional enrichment analysis of target genes are therefore significant ways by which the functions of miRNAs identified in the present study may be explained. The potential target genes of all the differentially expressed miRNAs in four groups were predicted using RNAhybrid (28), MiRanda (29), and TargetScan (30), and the results from the three algorithms were intersected. Subsequently, the Gene Ontology (GO) (http://www.geneontology.org/) and the Kyoto Encyclopedia of Genes and Genomes (KEGG) (http://www.genome.jp/kegg/) were used to recognize the main biological functions and identify functional pathways.

### The miRNA-mRNA interaction network analysis

The differentially expressed miRNAs were analyzed in targeted association with the 984 mRNAs screened in our previous study (31), pairs with Pearson correlation coefficient < −0.7 and *p*-value < 0.05 were selected as co-expressed negative miRNA-mRNA pairs and a miRNA-mRNA interaction network was constructed. The interaction analysis was visualized by Cytoscape software (32).

## Results

### The serum T, E_2_, FSH, and LH concentrations

**Figure 1:**
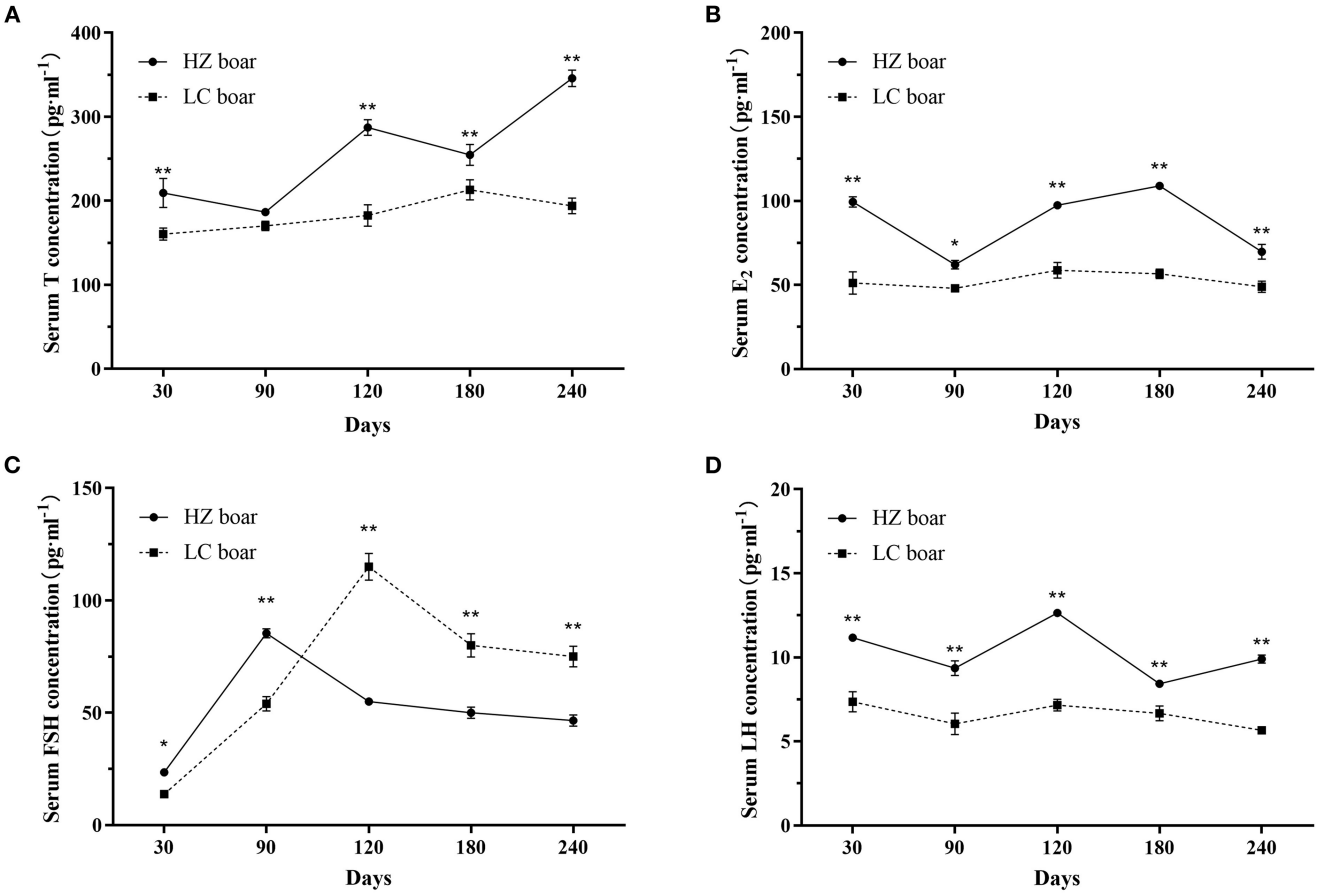
Serum sex hormone concentration in HZ and LC boar at different ages. **(A)** Serum T concentration; **(B)** Serum E_2_ concentration; **(C)** Serum FSH concentration; **(D)** Serum LH concentration; The data are expressed as the Mean ± SD, *n* = 3 per group. *p < 0.05 and **p < 0.01.

The serum T, E_2_, FSH, and LH concentrations were compared in HZ and LC boars at five different ages. Results showed that HZ boars had significantly higher serum *T* concentrations than LC boars at 30 (209.53 ± 17.25 vs. 160.44 ± 7.08 pg·ml^−1^), 120 (287.27 ± 9.10 vs. 182.59 ± 12.81 pg·ml^−1^), 180 (254.71 ± 12.49 vs. 213.02 ± 12.09 pg·ml^−1^), and 240 (345.92 ± 10.02 vs. 194.07 ± 9.32 pg·ml^−1^) days of age (*p* < 0.01, Figure 1A). Serum FSH was remarkably higher than that of LC boars at 30 (23.46 ± 1.25 vs. 13.76 ± 0.94 pg·ml^−1^) and 90 (85.34 ± 3.58 vs. 54.09 ± 3.14 pg·ml^−1^) days of age, and lower than that of LC boars at 120 (55.07 ± 2.78 vs. 115.04 ± 6.10 pg·ml^−1^), 180 (50.13 ± 4.36 vs. 80.04 ± 5.20 pg·ml^−1^), and 240 (46.55 ± 4.29 vs. 75.11 ± 4.58 pg·ml^−1^) days of age (*p* < 0.05/0.01, Figure 1C). Serum E_2_ and LH concentrations were higher than those of LC boars at all ages (*p* < 0.05/0.01, Figures 1B,D).

### Histological analysis of the testes of HZ and LC boars

**Figure 2:**
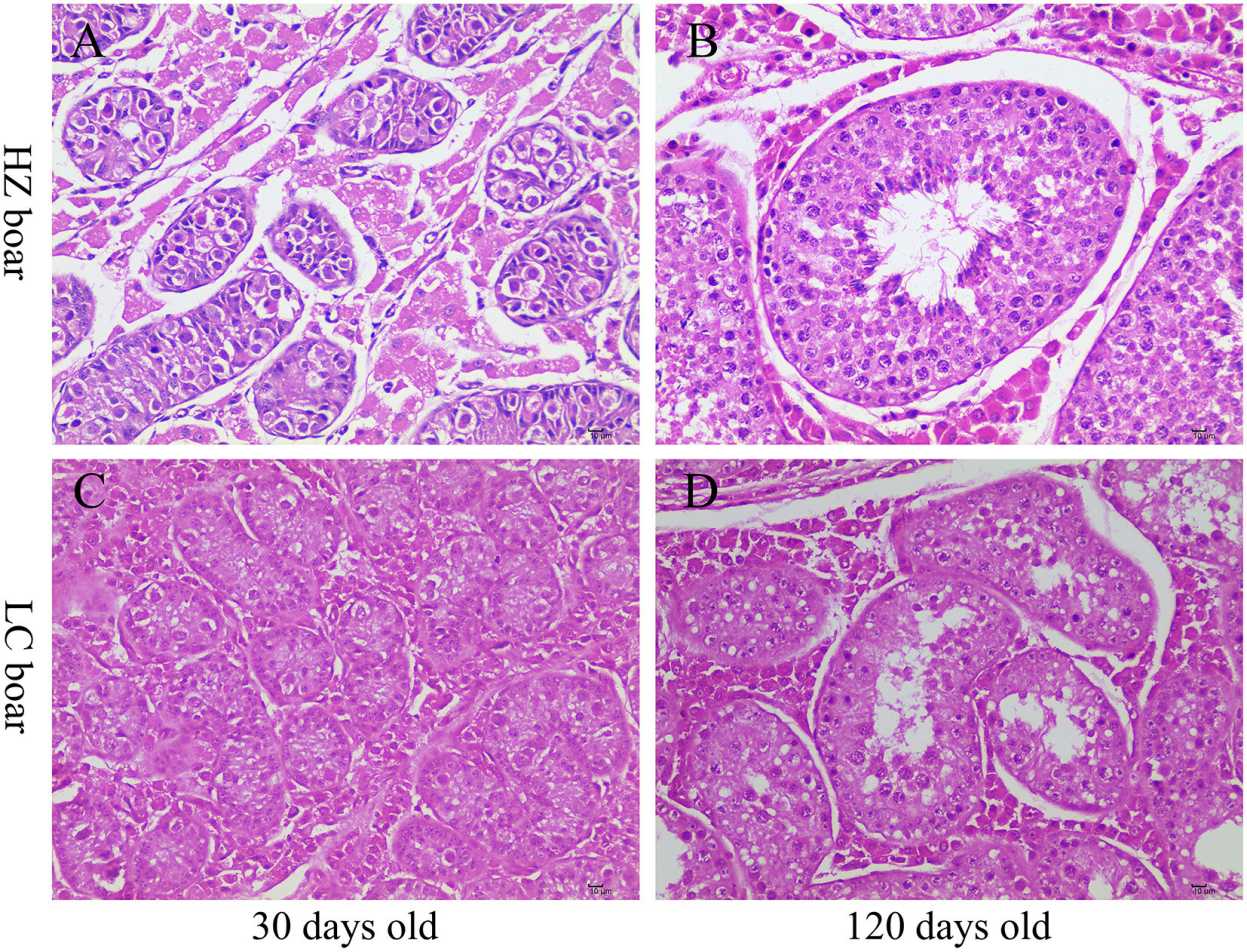
Histological observation of testis tissue in HZ and LC pigs at 30 and 120 days of age. **(A-D)** Represent the testicular cross-sections of 30-days-old HZ, 120-days-old HZ, 30-days-old LC, and 120-days-old LC boar at 400 × magnification, respectively.

Histological analysis showed that the diameter and lumen area of the seminiferous tubules increased significantly with age. Although few spermatogonia and Sertoli cells were found in 30-days-old HZ and LC boars testes tissues, numerous spermatogonia were observed in the HZ testes tissues. Spermatogenic cells were more abundant in type and number in 120-days-old HZ boars compared to LC boar testes, and mature spermatozoa were observed (Figure 2, Table 1). The results showed that HZ boars had earlier testicular development than LC boars and had reached sexual maturity at 120 days of age.

**Table 1:** Diameter of seminiferous tubules in testes of Hezuo boar and Landrace boar of different ages, number of spermatogenic cells and Sertoli cells (mean ± SEM).

**Parameters**	**Hezuo boar (HZ)**		**Landrace boar (LC)**	
	**30-days-old**	**120-days-old**	**30-days-old**	**120-days-old**
Diameter of spermatogenic tubules (μm)	80.85 ± 4.83^b^	176.37 ± 17.31^a^	56.53 ± 2.55^b^	152.11 ± 2.88^a^
Number of spermatogenic cells	21.78 ± 1.09^c^	149.78 ± 3.61^a^	15.00 ± 1.58^c^	98.89 ± 3.95^b^
Number of Sertoli cells	1.78 ± 0.44^b^	8.44 ± 0.91^a^	2.11 ± 0.22^b^	4.75 ± 0.91^a^

In each row, values with different superscripts are significantly different (P < 0.05).

### The summary of small RNA sequencing data

**Figure 3:**
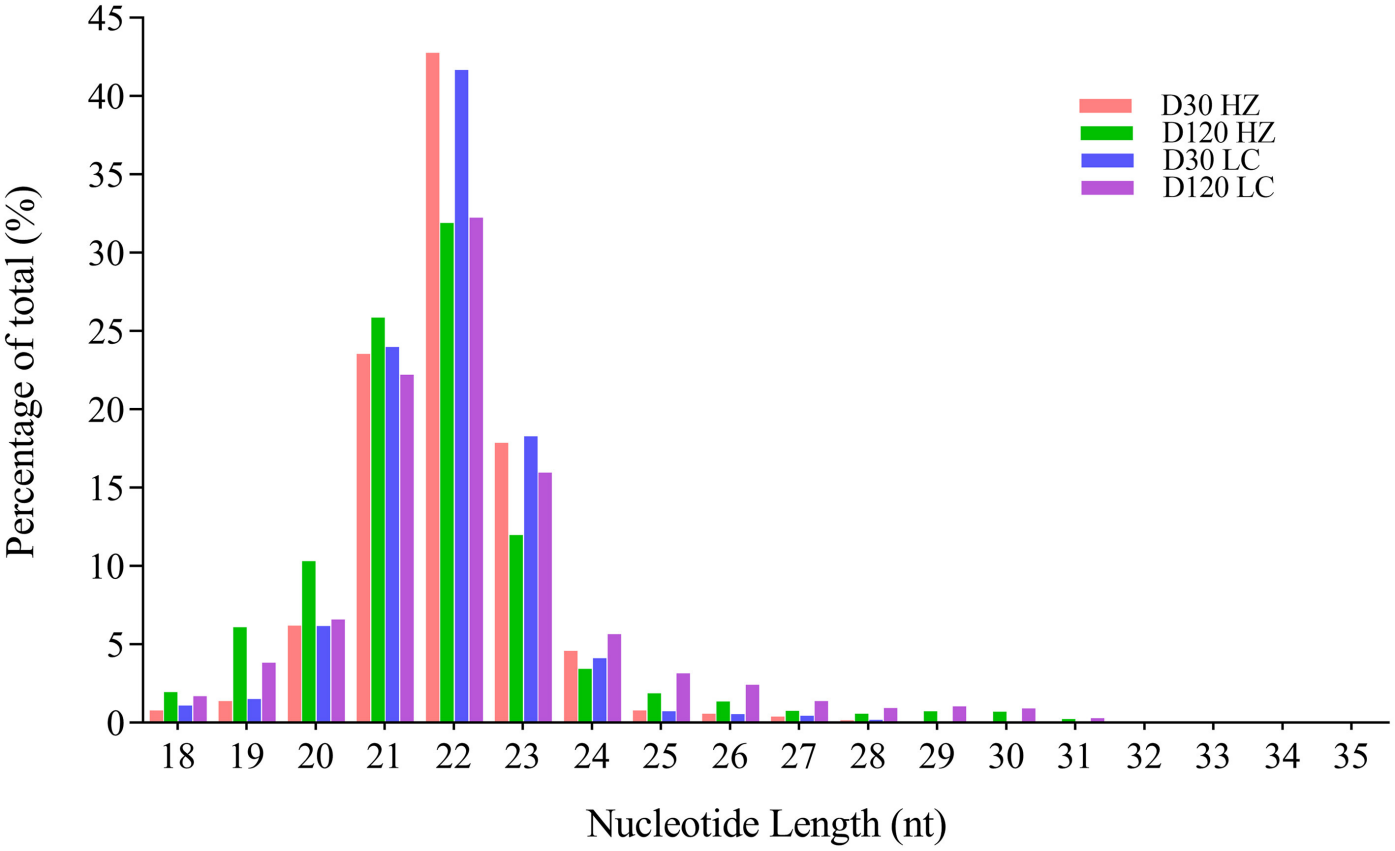
Length distribution and percentage of total tags in the small RNA libraries.

A total of 34997505, 33597522, 45582226, and 51209280 clean reads were generated in testis from 30-days-old HZ boar, 120-days-old HZ boar, 30-days-old LC boar, and 120-days-old LC boar, respectively. After eliminating the low quality reads and adaptor sequences, 34727584, 33260100, 45235416, and 50707768 clean tags were obtained and used for further analysis (Supplementary Table 2). After comparing the small RNA sequences with GenBank and Rfam, we removed known types of RNA sequences including rRNA, small cytoplasmic RNA (scRNA), small nuclear RNA (snRNA), small nucleolar RNA (snoRNA), and tRNA. A total of 54172719 and 75599762 clean tags from HZ and LC boar testes were mapped to the porcine reference genome (*Sus scrofa* 11.1), respectively (Supplementary Table 3). Those tags that were mapped to exons, introns and repetitive sequences were also removed. The length distribution of small RNA was similar in the testes of both breeds during the two periods (Figure 3). Most of the small RNA was from 21 to 24 nt and miRNA of 22 nt in length was the most common in length, accounting for 42.78, 31.90, 41.67, and 32.25% from 30-days-old HZ boar, 120-days-old HZ boar, 30-days-old LC boar, and 120-days-old LC boar, respectively. This was followed by miRNA of 21 nt length (23.56, 25.87, 24.01, and 22.21%, respectively) and 23 nt length (17.88, 12.00, 18.30, and 15.99%, respectively).

### Identification of exist, known and novel miRNA

**Figure 4:**
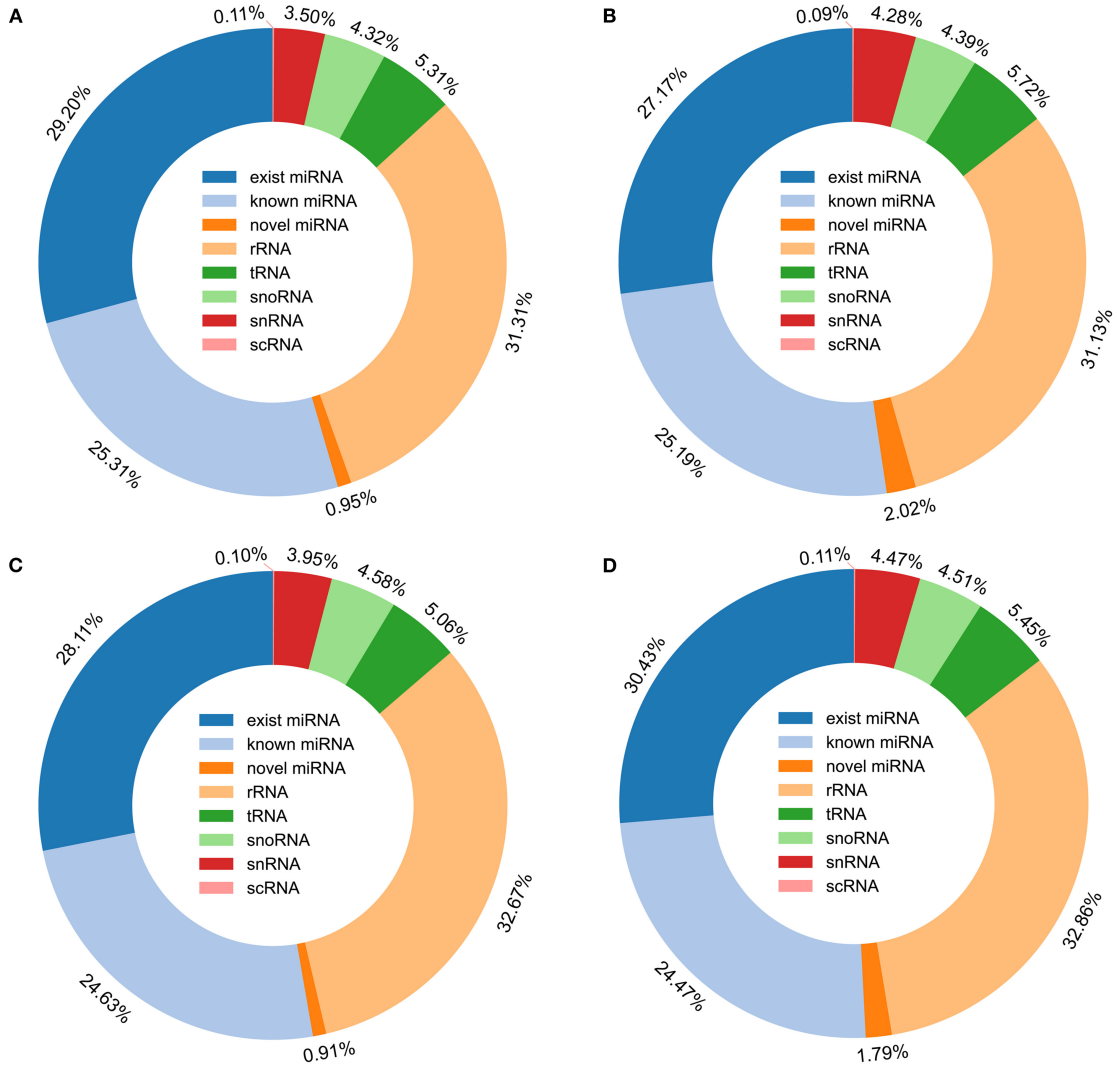
Distribution of small RNA in porcine testis. **(A)** Small RNA type in testis of 30-days-old HZ boar; **(B)** Small RNA type in testis of 120-days-old HZ boar; **(C)** Small RNA type in testis of 30-days-old LC boar; **(D)** Small RNA type in testis of 120-days-old LC boar.

The remaining tags were then mapped to porcine miRNA (exist miRNA and known miRNA) in miRbase 22.1, a total of 359 exist miRNA and 761 known miRNA were found to be expressed of the two breeds during two periods. All tags, that were not annotated to miRbase 22.1, were used to predict novel miRNA according to their genome positions and hairpin structures predicted by mirdeep2, a total of 332 miRNA was determined to be novel miRNAs (Supplementary Table 4). In all the annotated small RNA, miRNA including existing miRNA, known miRNA, and novel miRNA were the most abundant, and accounting for 55.46, 54.38, 53.65, and 56.69% of the number of small RNA unique tags from 30-days-old HZ boar, 120-days-old HZ boar, 30-days-old LC boar, and 120-days-old LC boar, respectively (Figure 4).

**Figure 5:**
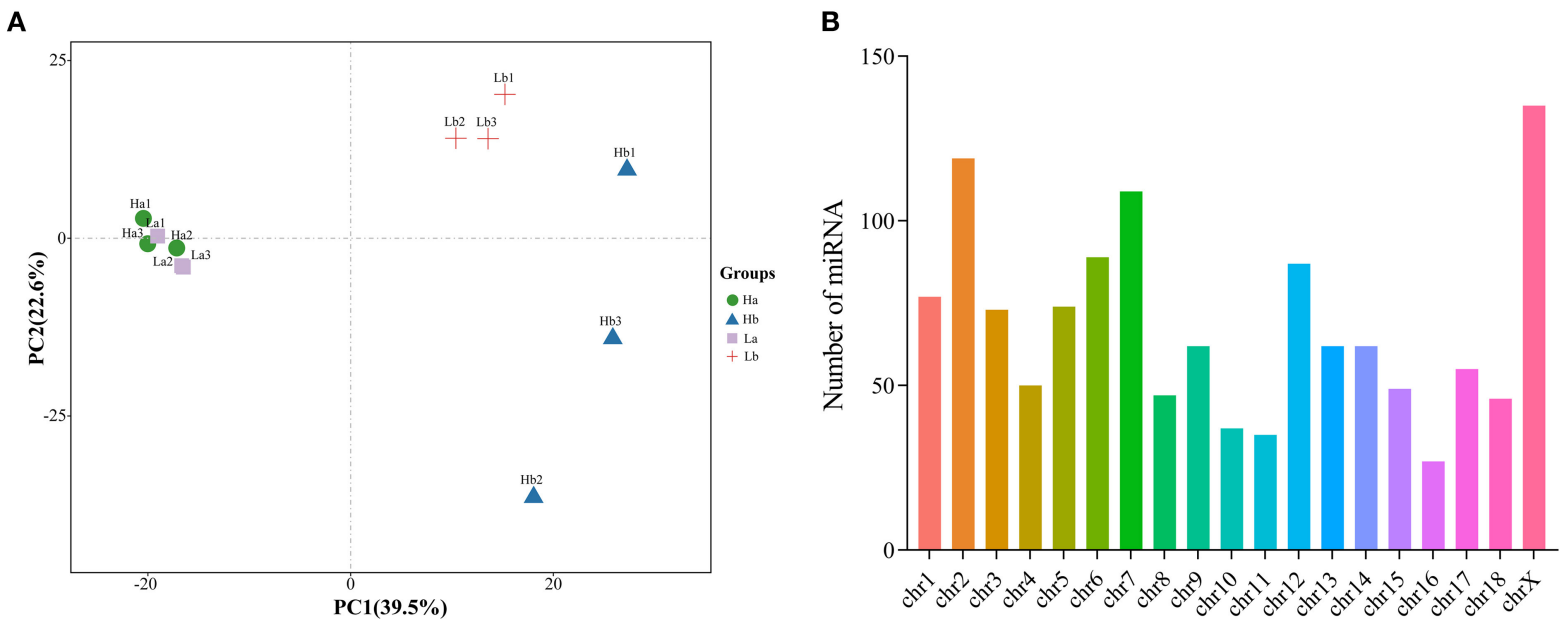
Component analysis of miRNA in porcine testis. **(A)** PCA plot of miRNA expressed in each sample; **(B)** Distribution of miRNA on porcine chromosome.

Based on normalized all miRNA count, principal component analysis (PCA) was conducted. These results showed that the six samples for the Ha and Lb group were close, while the six samples at Hb and Lb were more remote. miRNA expression was distinct between during two periods, and was different in 120-days-old HZ and LC boar (Figure 5A). Moreover, all miRNA was mapped to porcine chromosomes by blasting with the porcine reference genome. Over 89% of miRNA could be perfectly mapped to the porcine genome; however, about 11% could not be found on the porcine genomic sequence. The mapped miRNA was mainly distributed on Chr 2, 7, 12 and X (Figure 5B).

### Differentially expressed (DE) miRNA in testes of both porcine breeds during two periods

**Figure 6:**
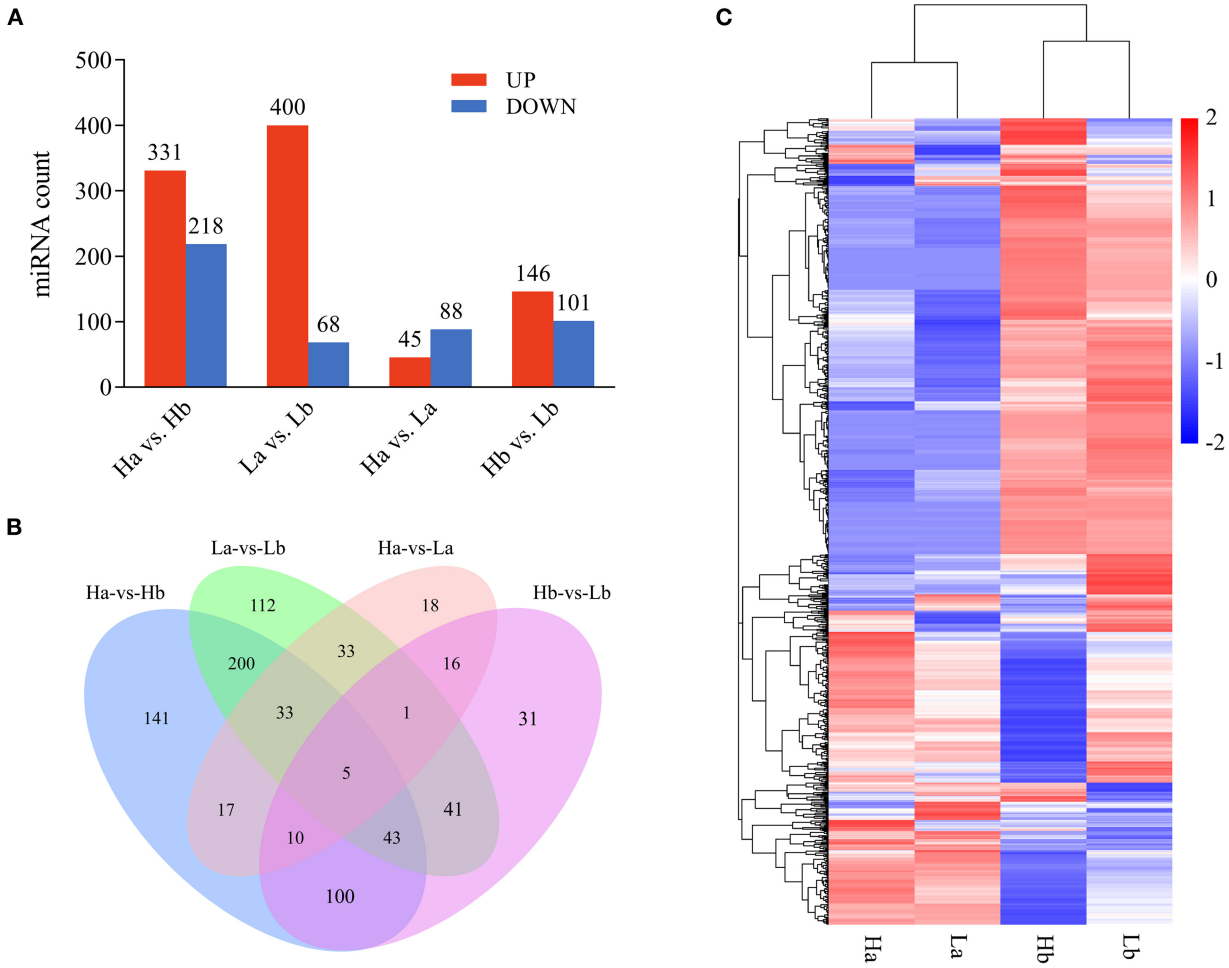
Differentially expressed profiles of miRNA. **(A)** The number of differential expressed miRNA in each analysis group; **(B)** Venn diagram of differential expression of miRNA between Ha vs. Hb, La vs. Lb, Ha vs. La, and Hb vs. Lb; **(C)** Hierarchical clustering of miRNA expression. miRNA profiles in the testes of both breeds during the two periods were clustered.

To investigate the DE miRNAs, the expression levels of the miRNA was first normalized to obtain the expression levels of TPM. The DE miRNA was selected using the edgeR software, when the |fold change| > 2 and *p*-value < 0.05. A total of 331 up- and 218 down-regulated, 400 up- and 68 down-regulated, 45 up- and 88 down-regulated, and 146 up- and 101 down-regulated miRNA were detected in Ha vs. Hb, La vs. Lb, Ha vs. La and Hb vs. Lb, respectively (Figure 6A, Supplementary Table 5). Venn diagram was generated using these datas and showed that 5 miRNAs (ssc-miR-1249, ssc-miR-153, miR-2137-z, miR-127-z, and miR-21-z) were shared among these four groups (Figure 6B). These results demonstrated that miRNA was differentially expressed in both breeds during the two stages and may play special roles in sexual maturation. DE miRNA was divided into four groups with 12 libraries, while miRNA expression was similar compared to the same period (Ha and La, Hb and Lb), but differentially expressed in different periods (Ha and Hb, La and Lb) in both breeds (Figure 6C).

### RT-qPCR validation of the DE miRNA

**Figure 7:**
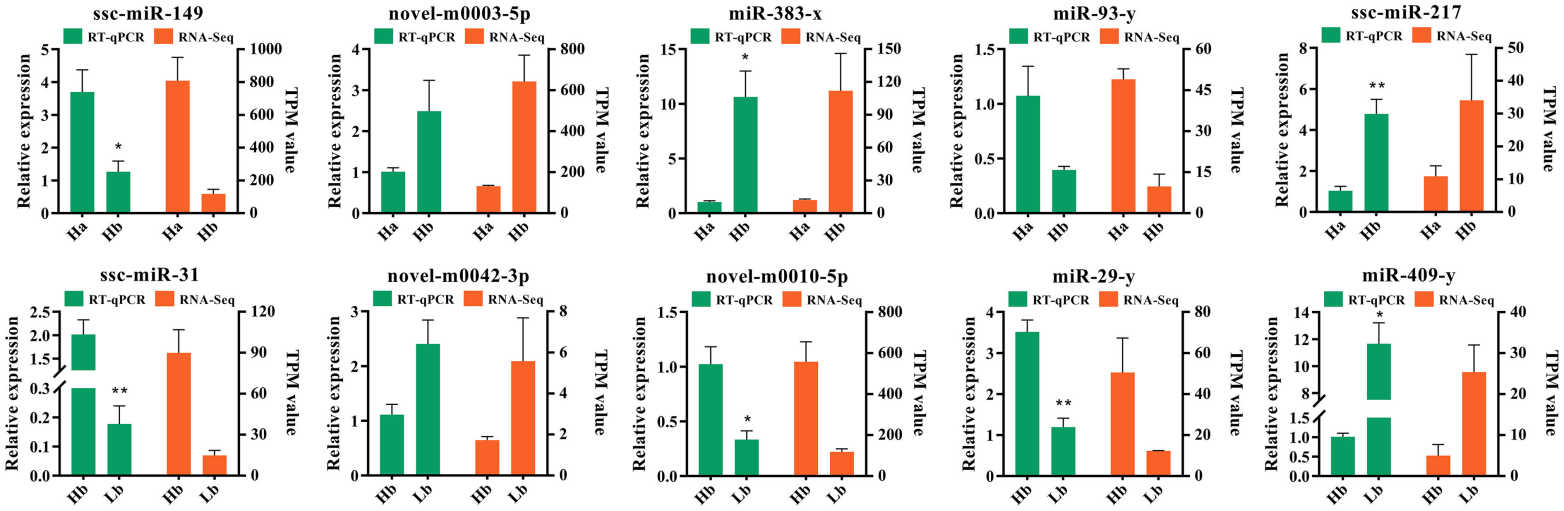
Validation of 10 differentially expressed miRNAs by RT-qPCR. Left Y-axis (green) shows the relative expression levels of miRNAs using RT-qPCR, whereas the right Y-axis (orange) shows the TPM values of the miRNAs using small RNA-Seq. ^*^Indicates *p* < 0.05, ^**^indicates *p* < 0.01. Data are shown as means ± SEM. Three biological replicates were used.

In order to validate authenticity of the miRNA sequencing datas, 10 DE miRNAs were randomly selected, ssc-miR-149, ssc-miR-217, ssc-miR-31, miR-383-x, miR-29-y, miR-93-y, miR-409-y, novel-m0010-5p, novel-m0003-5p, and novel-m0042-3p, to perform the RT-qPCR. These miRNA regulatory orientation and expression patterns were in agreement with their sequencing results (Figure 7), in which 5 miRNAs (ssc-miR-217, miR-383-x, miR-409-y, novel-m0003-5p, and novel-m0042-3p) were up-regulated and 5 miRNAs (ssc-miR-217, ssc-miR-31, miR-29-y, miR-93-y, and novel-m0010-5p) were down-regulated. Thus the miRNA sequencing datas were reliability and repeatability for detecting the DE miRNAs.

### Target gene prediction and enrichment analysis

Predicting the target gene of miRNA is important because they regulate biological processes by suppressing the translation of target gene. Thus, to further understand the physiological and molecular functions of DE miRNAs, miRanda, and TargetScan were used to predict target gene of total DE miRNA. A total of 5085247 binding sites were identified with all 801 DE miRNAs and 59508 target genes were predicted. To predict the function of the DE miRNAs, GO analysis was performed with their target genes. A total of 99, 94, 323, and 229 highly enriched GO terms in biological processes, cellular components, and molecular functions were derived from Ha vs. Hb, La vs. Lb, Ha vs. La and Hb vs. Lb groups, respectively (*p*-value < 0.05, Supplementary Table 6). Most enriched GO terms in four comparison groups included cellular developmental process (GO: 0048869), cell morphogenesis (GO: 0000902), metabolic process (GO: 0008152), neuron development (GO: 0048666), animal organ development (GO: 0048513), and developmental process involved in reproduction (GO: 0003006). These DE miRNA target genes are involved in cell development, metabolism, regulation of organismal development and reproduction, and some other important biological functions. KEGG pathways analysis revealed that DE mRNAs were assigned to 129, 124, 119, and 143 pathways in Ha vs. Hb, La vs. Lb, Ha vs. La, and Hb vs. Lb groups, respectively (*p*-value < 0.05, Supplementary Table 7). These pathways included Wnt (ko04310), Rap1 (ko04015), Hippp (ko04390), PI3K-Akt (ko04151), mTOR (ko04150), MAPK (ko04010), and Ras (ko04014). These results indicated that DE miRNAs in the testes of HZ and LC boars were involved in testicular development and spermatogenesis through the above pathways.

### Integrated analysis between DE miRNA and target mRNA in HZ and LC boars during the two stages

**Figure 8:**
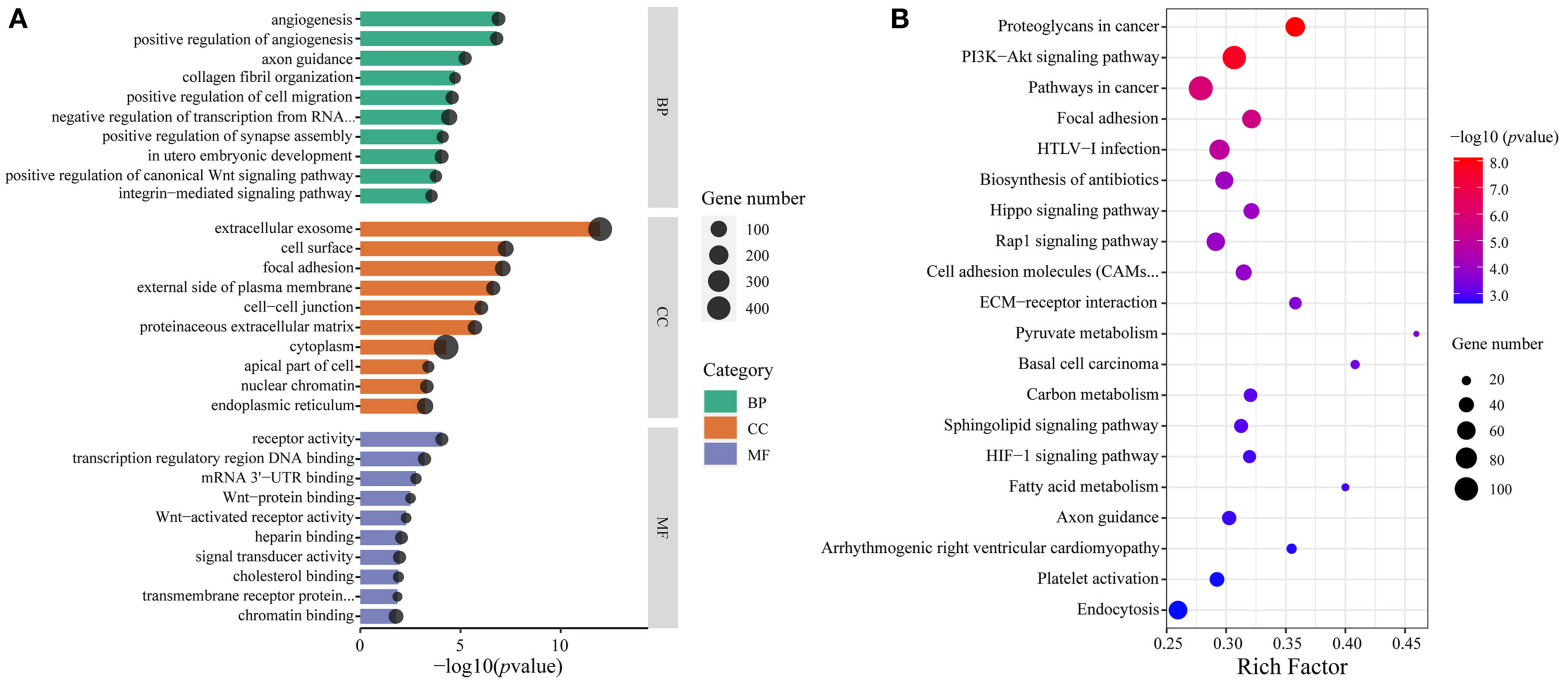
Functional enrichment analysis of candidate miRNAs target genes. **(A)** The top 10 GO terms for BP (green), CC (orange), and MF (purple), respectively. The x-axis indicates *p*-value and y-axis indicates the names of the cluster, and the size of dots indicates the numbers of genes; **(B)** The top 20 KEGG pathways of candidate miRNAs target genes. The x-axis indicates Rich Factor and the y-axis indicates name of KEGG pathway. The size of dots indicates the numbers of genes and the color of dots indicates *p*-value.

The mRNA expression data of 12 samples from our previous study were used for a pairwise integrated analysis (31). To explore miRNAs related to precocious puberty in HZ boars, we screened 158 DE miRNAs from the Ha and Hb, Hb and Lb groups. These miRNAs contained important information both during testicular development in HZ boars and differences of sexual maturity in HZ and LC boars. We performed targeted association of these candidate miRNAs with previously identified DE mRNAs and functional enrichment analysis. A total of 234 GO terms (132 BP, 48 CC, and 24 MF) and 70 KEGG terms were enriched (*p*-value < 0.05, Supplementary Table 8). These GO terms were in-volved in focal adhesion (GO: 0005925), vasculogenesis (GO: 0001570), mitochondrion (GO: 0005739), calcium ion binding (GO: 0005509) and male gonad development (GO: 0008584). Figure 8A showed the top 10 GO terms (according to *p*-value) for BP, CC, and MF, respectively. These signal pathways include PI3K-Akt (ssc04151), Hippo (ssc04390), Rap1 (ssc04015), Glycolysis/Gluconeogenesis (ssc00010), and Ras (ssc04014) signaling pathways. This implies that these molecules may play a key role in regulating precocious puberty through the above signaling pathways. The top 20 KEGG pathways (according to *p*-value) were shown in Figure 8B. This implies that these miRNAs may play an important regulatory role in precocious puberty by targeting these genes through the above mentioned signaling pathways.

### The miRNA-mRNA interaction network

**Figure 9:**
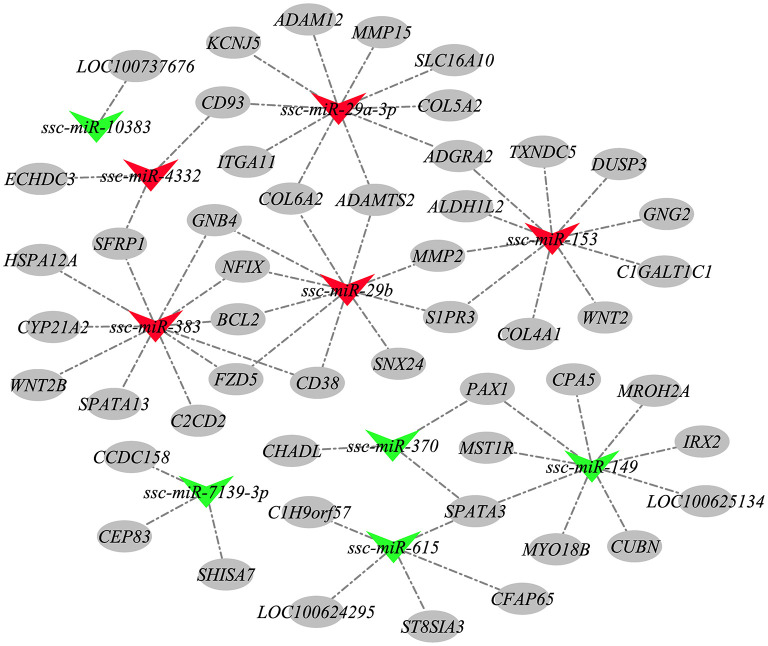
The miRNA-mRNA network. Nodes represent miRNA or target gene, and edges represent the negative regulatory interaction between miRNA and mRNA. Red triangles represent up-regulated miRNAs in Ha vs. Hb group and down-regulated in Hb vs. Lb group, green triangles represent miRNAs down-regulated in Ha vs. Hb group and up-regulated in the Hb vs. Lb group, and gray circles represent the target mRNAs of these miRNAs.

According to the negative regulatory effects of miRNAs on mRNAs, these 158 candidate miRNAs were targeted for association with 984 mRNAs previously screened to further explore the potential involvement of HZ boar precocious miRNAs. A total of 2,525 miRNA-mRNA relationship pairs were identified (Supplementary Table 9). To better understand the interactions between the miRNAs and their target genes, 10 most differentially expressed exist miRNAs and some corresponding target mRNAs were selected to construct a miRNA-mRNA network (Figure 9). The 10 miRNAs included the 5 most up-regulated miRNAs (ssc-miR-153, ssc-miR-29a-3p, ssc-miR-29b, ssc-miR-4332, and ssc-miR-383) in Ha vs. Hb group and down-regulated in Hb vs. Lb group, and the 5 most down-regulated miRNAs (ssc-miR-7139-3p, ssc-miR-615, ssc-miR-10383, ssc-miR-370, and ssc-miR-149) in Ha vs. Hb group and up-regulated in the Hb vs. Lb group. These results implied that the interaction between miRNA and mRNA may be involved in precocious puberty.

## Discussion

The reproduction performance of sire is crucial in livestock production, which can bring higher economic benefits. Early sexual maturity is an excellent reproductive characteristic of some local pig breeds in China, such as Hezuo (HZ) pig. It can improve the utilization of female animals, shorten the generation interval of excellent females, and accelerate the genetic breeding process. Our previous works have shown that HZ boars have reached sexual maturity at 4 months of age (31). To explore potential regulatory mechanisms of early sexual maturation in HZ boars, the serum hormones (T, E_2_, FSH, and LH) of HZ and LC boars at 30, 90, 120, 180, and 240 days were measured and compared, and the miRNAs in the testes of HZ and LC boars at 30 and 120 days were identified and comparatively analyzed using small RNA-Seq.

The concentration of sex hormones is closely associated with the reproductive performance of boars, which can reflect the development of their reproductive organs in a certain extent. As expected, hormone assay results showed that the serum *T* levels were generally on the rise and were higher in HZ boars than in LC boars at all ages, which is consistent with other local pig breeds [such as Xiang pig (33) and Erhualian pig (34)]. The secretion of T is mainly influenced by LH secreted by pituitary gland, and increases with the rise of LH (35). The trend of serum LH content was generally consistent with that of testosterone, indicating that LH can stimulate testicular Leydig cells to synthesize and secrete testosterone to promote spermatogenesis, and the increase in T levels were associated with accelerated postnatal division of testicular Leydig cells (36). Thus, the interaction of high testosterone and luteinizing hormone levels in HZ boars may have stimulated testicular development and spermatogenesis to reach sexual maturity more rapidly compared to LC boars. While *T* appears and acts on the male reproductive system, E_2_ also plays the same role for all target organs, working together with other hormones to perform the genesis, growth, maturation and regulation of the testes and germ cells (5). The serum E_2_ levels of HZ boars were higher than those of LC boars at all ages, indicating that high levels of E_2_ also play a regulatory role in sexual maturation. Interestingly, the serum FSH of HZ boars was higher than that of LC boars at 30 and 90 days, but significantly lower after 120 days. FSH promotes testicular Sertoli cell synthesis and stimulates the development of seminaferous epithelium and spermatogenesis (37). It may imply that the high levels of FSH play important regulatory functions in testicular development and spermatogenesis in HZ boars, especially before sexual maturation.

miRNA regulation is critical and effective mechanism underlying testicular development and spermatogenesis. miRNA has the potential to inhibit protein expression and degrade mRNA expression in germ cell proliferation and development in a post-transcriptional way (38), suggesting that as boars sexually mature, a certain number of some miRNA transcripts are needed to regulate processes related to sexual development. Read length distribution of 12 libraries showed that 21 to 24 nt represented the length of most small RNAs, of which 22 nt represented the highest percentage. This finding is consistent with the normal size of miRNAs reported in previous studies (39, 40).

Many mammalian miRNAs play important roles in development and other biological processes, and the expression patterns of miRNAs are tissue-specific or developmental stage-specific (41). Bioinformatics analysis of miRNAs derived from four pairwise comparisons (Ha vs. Hb, Ha vs. La, La vs. Lb, and Hb vs. Lb) from two different periods in HZ and LC boars showed that Ha and La were clustered and that Hb and Lb were clustered together (Figure 6C). These findings are consistent with our previous mRNA sequence analysis (35). However, the miRNA expression patterns of Hb and Lb were significantly different than that of Ha and Lb. These results may imply that the characteristics of HZ boars that have reached sexual maturity at 120 days are different from those of LC boars that have not yet reached puberty at the same age. A large number of miRNAs were up-regulated in Ha and La, but down-regulated in Hb and Lb.

To further identify miRNAs related to precocious puberty traits in HZ boars, we focused on two comparison groups (Ha vs. Hb and Hb vs. Lb) to screen for co-differentially expressed miRNAs. A total of 158 miRNAs were initially screened out for subsequent focused analysis. Based on our previous RNA-Seq data, we predicted target genes for these miRNAs and performed GO and KEGG analysis on these target genes. The results showed that most of them were involved in metabolism of various materials and were enriched in some common pathways (such as PI3K-Akt, Hippo and Rap1), which play a functional role in testis development and spermatogenesis (42–44). By further screening these miRNAs based on fold change and expression, ssc-miR-29b, ssc-miR-199b, ssc-miR-383, ssc-miR-149, ssc-miR-615, and ssc-miR-370 were identified. This suggests that these differentially expressed miRNAs might play important roles in the process of sexual maturation in HZ boars.

miR-199b and miR-29 are spermatogonial stem cells specific miRNAs (45). miR-199b was one of the 30 most abundantly expressed miRNAs in testicular and ovarian tissues (46) and was down regulated in mature porcine testis compared with immature testis (47), this is consistent with our results. In addition, miR-29b down-regulation was reported to confirm modulated methylation of genomic DNA in primordial germ cells by targeting *Dnmt3a* and *Dnmt3b* essential for gonadal development (48). The up-regulation of ssc-miR-29b expression in sexually mature HZ boars in our study was likely related to its role in testicular development and spermatogenesis, and results also showed that ssc-miR-29b targets multiple collagen genes such as *COL1A2, COL4A1*, and *COL6A2*. *COL6A2* has been reported to play a major role in cell adhesion and affects the secretion of testicular Leydig cells (49). Interestingly, we also found that ssc-miR-29b targets the *ITGB1* gene and involved in gametogenesis and cell adhesion molecules. *ITGB1* can also activate the PI3K-Akt signaling pathway (50), which plays a key role in embryonic testis cord formation (51) and the self-renewing division of SSCs (52). miR-383 is mainly localized to spermatogonia and primary spermatocytes in early spermatogenesis, and its expression has been reported to be down-regulated in the testes of patients with non-obstructive azoospermia (53), it has also been reported that miR-383 is associated with spermatogenesis as its targets a tumor suppressor, interferon regulatory factor-1 (54). Our study showed that miR-383 expression was upregulated in sexually mature HZ boars and it targeted *BCL2* and *CYP21A1*. In previous literature, *BCL2* is predicted by miR-449 and mediates spermatogonia apoptosis. High levels of BCL2 protein in male germ cells result in highly abnormal adult spermatogenesis with infertility (55). *CYP21A2* is responsible for 21 hydroxylase activity, mutations in *CYP21A2* can lead to 21 hydroxylase deficiency (56). It has been speculated in our previous studies to play roles in premature maturation of HZ boars (31). Thus, we suggested that miR-383 may target *BCL2* and *CYP21A2* to play a regulatory role in precocious puberty in HZ boars. Notably, our results indicated that ssc-miR-149, ssc-miR-370 and ssc-miR-615 are highly expressed in immature HZ boars and simultaneously target the *SPATA3* gene. *SPATA3* has been demonstrated to be the most significantly down-regulated gene in the testes of infertile patients (57) and to play a key role in mouse spermatogonia development (58). Moreover, our previous study has been speculated that it may also play important roles in precocious puberty of HZ boars (31). Hence, we further speculated that ssc-miR-149, ssc-miR-370, and ssc-miR-615 co-regulate *SPATA3* gene to play important roles in precocious puberty of HZ boars.

## Conclusions

In this study, we compared the serum T, E_2_, FSH, and LH levels of HZ and LC boars at different ages (30, 90, 120, 180, and 240 days). High levels of sex hormones may be correlated with the precocious development of HZ boars. Small RNA-Seq was used to identify the miRNA of testes in HZ boars and LC boars at 30 and 120 days. Based on our previous RNA-Seq data, a joint analysis was performed to initially screen out some miRNAs (ssc-miR-29b, ssc-miR-199b, ssc-miR-383, ssc-miR-149, ssc-miR-615, and ssc-miR-370) that may regulate precocious sexual maturation traits in HZ boars. The study provides an improved understanding of the roles of miRNAs in precocious sexual maturation in HZ boars.

## Data availability statement

The datasets presented in this study can be found in online repositories. The names of the repository/repositories and accession number(s) can be found in the article/Supplementary material.

## Ethics statement

The animal study was reviewed and approved by Faculty Animal Policy and Welfare Committee of Gansu Agricultural University. Written informed consent was obtained from the owners for the participation of their animals in this study.

## Author contributions

BZ, ZY, and SG designed the study. BZ performed the research and analyzed the data with the support of ZY in statistics and prepared and wrote the original draft. ZY, YG, JL, ZW, PW, QY, XH, and SG supervised the project. ZY and SG reviewed the paper. All authors contributed to the article and approved the submitted version.

## Funding

This work was supported by the Breeding and Cultivation of New Local Pig Breeds (Synthetic Lines) in the Northern Region (2021YFD1301200) and the Protection and Quality Improvement of Gansu Local Pig Germplasm Resources (GSLK-2021-13).

## Conflict of interest

Author YG was employed by Jilin Rongtai Agricultural Development Co., Ltd., China. The remaining authors declare that the research was conducted in the absence of any commercial or financial relationships that could be construed as a potential conflict of interest.

## Publisher's note

All claims expressed in this article are solely those of the authors and do not necessarily represent those of their affiliated organizations, or those of the publisher, the editors and the reviewers. Any product that may be evaluated in this article, or claim that may be made by its manufacturer, is not guaranteed or endorsed by the publisher.
